# Passive Temperature Stabilization of Silicon Photonic Devices Using Liquid Crystals

**DOI:** 10.3390/ma7032229

**Published:** 2014-03-14

**Authors:** Joanna Ptasinski, Iam-Choon Khoo, Yeshaiahu Fainman

**Affiliations:** 1 Department of Electrical and Computer Engineering, University of California San Diego, La Jolla, CA 92037, USA; E-Mail: fainman@ece.ucsd.edu; 2 Space and Naval Warfare Systems Center Pacific, San Diego, CA 92152, USA; 3 Electrical Engineering Department, Pennsylvania State University, University Park, PA 16802, USA; E-Mail: ick1@psu.edu

**Keywords:** liquid crystals, thermal stabilization, thermo-optic coefficient, silicon photonics, ring resonator, passive tuning

## Abstract

In this work we explore the negative thermo-optic properties of liquid crystal claddings for passive temperature stabilization of silicon photonic integrated circuits. Photonic circuits are playing an increasing role in communications and computing, but they suffer from temperature dependent performance variation. Most existing techniques aimed at compensation of thermal effects rely on power hungry Joule heating. We show that integrating a liquid crystal cladding helps to minimize the effects of a temperature dependent drift. The advantage of liquid crystals lies in their high negative thermo-optic coefficients in addition to low absorption at the infrared wavelengths.

## 1. Introduction

Silicon photonic devices and circuits offer a rapidly growing and promising technology for high-speed signal transmission systems with data rates of 100 Gbps, which far exceed the capabilities of copper cabling. Such devices are suited for data centers and high performance computing applications where standard copper based Ethernet networking is inadequate. Photonics based systems offer the advantage of reduced energy consumption in addition to the ability to pack a larger number of communication channels into a smaller space [1]. In the next few years, large numbers of silicon photonic products will come to market and there will be an increase in the number of complex silicon photonic systems developed in academia [2]. As silicon photonic chips mature, the technology is likely to be increasingly used in processing tasks such as interconnecting multiple cores within processor chips to boost access to shared cache and busses. Eventually, silicon photonics may be involved in actual processing, augmenting or replacing a chip’s semiconductor transistors with optical equivalents with greater computing performance [3]. The field of silicon photonics is expressly well positioned at this time as paths for commercialization are now widely accessible, the costs and risks associated with prototyping products have dropped, and adopting the same silicon processing tools that the semiconductor industry uses to fabricate Complementary Metal–Oxide–Semiconductor (CMOS) transistors opens access to an immense infrastructure for yield improvement, metrology and process control [2]. Conversely, as optics penetrates deeper into the chip temperature stability becomes more important due to silicon’s high thermo-optic coefficient (1.86 × 10^−4^/°C) accompanied by an appreciable modification of the refractive index in the presence of rising temperature and resulting in performance deterioration of photonic devices and systems [4,5]. Correspondingly, at power densities of 100 W/cm^2^ in modern microelectronic Very Large Scale Integration (VLSI) chips, the problem of heat dissipation is a major challenge even with the most advanced packaging technologies. Local temperature stabilization becomes impossible with thousands of devices with varying temperature profiles across a single chip [6].

There has been a tremendous amount of research on the suppression of temperature sensitivity in silicon based chipscale devices and a common approach consists of using external heaters or thermoelectric coolers. A related scheme focuses on the use of direct local heating of the silicon device by alternating the bias current, or using silicon itself as a resistive material for heating [4,7]. However, as these approaches are active, they increase power consumption and account for the largest share in a power budget of state-of-the-art silicon photonics [4], in addition to demanding a large device foot-print and cost. Passive thermal stabilization techniques rely on the use of a negative thermo-optic coefficient (TOC) material to offset silicon’s high positive TOC [5,6], tailoring the degree of optical confinement in silicon waveguides [7], or a careful design of the device geometry [4,8,9]. Materials used for thermal stabilization consist of polymers, such as acrylates demonstrated by Ye *et al.* [10], where a temperature dependent peak resonance wavelength shift of a racetrack resonator was reduced 8.3 times. However, the demonstration was geometry specific and performed over a very small temperature range. Other research consisted of working with Polymethyl methacrylate, a common lithography resist, or ExguideTM LFR-372 (ChemOptics Inc., Daejeon, Korea) over a wider temperature range with similar findings [5,8,11]. A drawback to polymers is that they are vulnerable to temperature degradation, chemical instability, ultraviolet (UV) aging, and poor mechanical characteristics [12,13]. Alternative methods which rely on engineering of the device geometry to lower the temperature sensitivity of the entire waveguiding system require additional space on the chip, are sensitive to fabrication tolerances and assume that the temperature compensating devices are located in the same thermal hotspot [7,12].

Here we explore a passive thermal stabilization scheme for resonant photonic devices using liquid crystal (LC) claddings. Liquid crystals’ relatively low viscosity (the viscosity of E7 is 40 cps at 20°C [14]) makes it possible to backfill them into chambers made during the fabrication process in a manner similar to microfluidic devices [15–17]. The main allure of liquid crystal claddings lies in their large negative thermo-optic coefficients and low absorption at the infrared and visible wavelengths, which translates into lower insertion losses. The thermo-optic coefficient d*n/*d*T* in nematics is extraordinarily large, ranking among the largest of all known materials [18]. The rod-like nematic liquid crystals exhibit optical birefringence: ordinary refractive index *n*_o_ for light polarized perpendicular to the liquid crystal and extraordinary refractive index *n*_e_ for light polarized parallel to the liquid crystal [19]. LC crystalline properties become apparent when the liquid crystal is contained in thin flat cells. The alignment of the liquid crystal axis in such cells is essentially controlled by the cell walls whose surfaces are treated in a variety of ways to achieve various director axis alignments [20]. Homeotropic alignment (where the LC long axis is perpendicular to the surface) is typically achieved by treating the cell walls with a surfactant such as hexadecyl-trimethyl-ammoniumbromide (HTAB) and planar alignment is most easily achieved by rubbing unidirectionally with a lens tissue; LCs then align their long axis along the rubbed direction [20]. As a material, LCs are low cost, easy to use (including the possibility of filling various volumes), offer high damage threshold to laser radiation, and overcome roughness and stress induced scattering loss and polarization dependence [21,22]. Moreover, LC molecular design provides leeway in modifying their structure and properties [18–22].

We use ring resonators, which are highly sensitive to changes in the refractive index to characterize the thermo-optic properties of liquid crystals. A ring resonator consists of a closed loop waveguide commonly in the shape of a ring or a racetrack. Coupling to and from the device is achieved by placing bus waveguides within a close proximity of the ring, allowing for evanescent modes to overlap and allow coupling [23]. The ring behaves as an interferometer and shows a resonance for light whose phase change after each full trip around the ring is an integer multiple of 2π, where the difference between the vacuum wavelengths corresponding to two resonant conditions is referred to as the free spectral range (FSR) [23,24]. A resonant wavelength change is observed in response to an effective index change for the resonant mode and the amount of the resonant wavelength shift is influenced by the length of the ring perimeter [23], where the resonant wavelength is described by:

$$\lambda_{\text{res}}=\frac{L\cdot n_{eff}}{m}$$ (1)

here *L* is the ring perimeter, *n*_eff_ is the effective index of the mode, and *m* is an integer.

**Figure 1.**
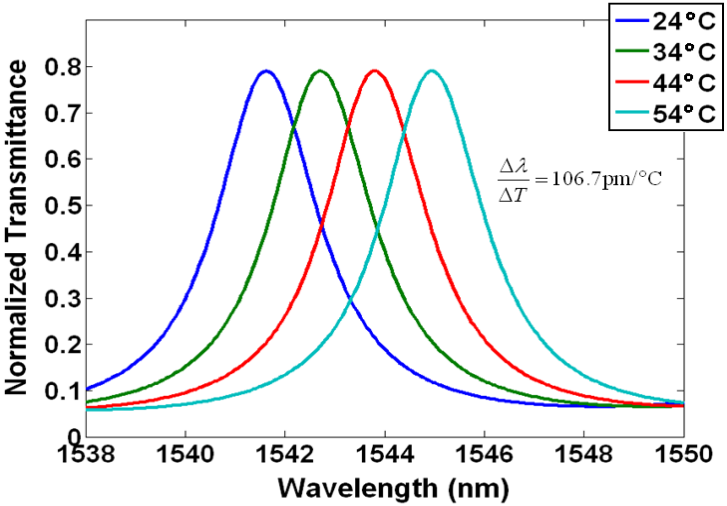
Simulation showing a resonance shift due to rising temperature for a ring resonator clad in silicon dioxide.

Figure 1 depicts a 2D finite-element simulation in COMSOL Multiphysics (Palo Alto, CA, USA) showing the projected fundamental TE like mode resonance shift due to rising temperature for a silicon ring clad in SiO_2_. Silicon photonic waveguides commonly consist of a silicon core and silica claddings, because the large refractive index contrast between the core and cladding allows for total internal reflection with a very large incident angle [24–26]. Typically, the TE like mode is more frequently used than the TM like mode due to its low bending loss, stronger confinement in the waveguide core, and minimal leakage into the silicon substrate beneath the buried oxide (BOX) layer. In this simulation, the ring was 500 nm wide, 250 nm tall, and with a perimeter of 62.2 μm. The effective index method was used in defining the effective mode indices and propagation constants of our ring resonator. The high TOC of silicon (Δ*n*_Si_/Δ*T* = 1.86 × 10^−4^/°C) together with the TOC of SiO_2_ (Δ*n*_SiO2_/Δ*T* = 1 × 10^−5^/°C) resulted in a 3.2 nm resonance shift for a 30 °C change in temperature, from 1541.7 nm to 1544.9 nm, which translates to Δλ/Δ*T* = 106.7 pm/°C.

In modern dense wavelength division multiplexing (DWDM) systems with channel spacing of <1 nm, a difference of Δλ/Δ*T* = 106.7 pm/°C will greatly influence channel location and crosstalk [27].

## 2. Results and Discussion

### 2.1. Liquid Crystal Materials

Liquid crystals used in the experiment consisted of 5CB (Sigma Aldrich, St. Louis, MO, USA), E7 (Merck, Hunterdon County, NJ, USA), Lixon ZSM-5970 (Chisso Corp., Minamata, Japan), and MDA-05-2968 (Merck). Table 1 depicts some of the common characteristics of these liquid crystal mixtures.

### 2.2. Results

**Figure 2.**
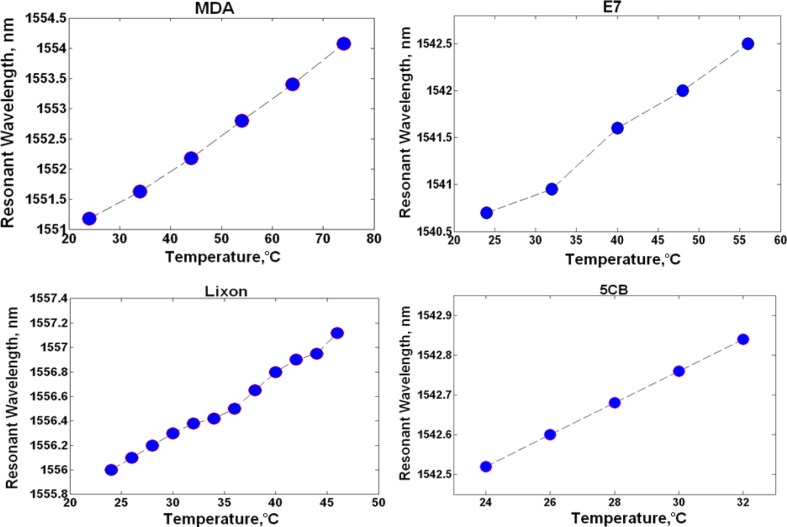
Measured ring resonator samples clad in liquid crystal (LC) mixtures.

**Figure 3.**
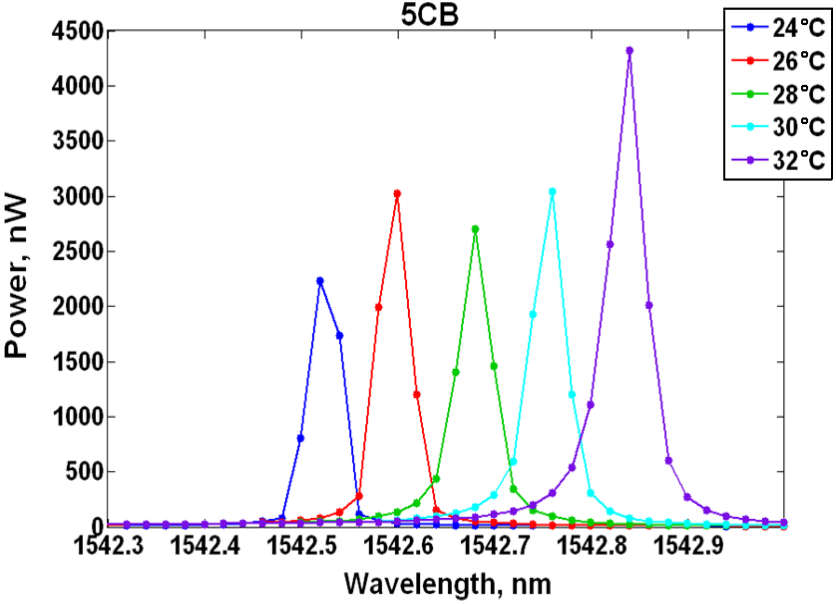
Peak wavelength shift for 5CB clad resonator. The thermal drift is 40 pm/°C.

Ring resonator samples consisting of 9.9 μm radius, 500 nm width, 250 nm height, 100 nm gap between the ring and bus waveguide were characterized. A measurement was performed on a sample clad in air, which resulted in an 87.5 pm/°C resonance shift and it served as the baseline. Subsequent measurements were completed with liquid crystal claddings listed in Table 1. It should be noted that the samples did not rely on an alignment layer in order to achieve a specific liquid crystal orientation and the liquid crystals were assumed to be randomly oriented exhibiting an average refractive index <*n*>. The experimental results are shown in Table 2 and Figure 2, where Table 2 provides a summary of the observed resonance shift per degree Celsius, while Figure 2 tracks the resonant wavelength change as a function of increasing temperature. MDA-05-2968 LC produced a peak wavelength shift of 58 pm/°C, while the best response was attained with 5CB (40 pm/°C) and it is further detailed in Figure 3, where the measured resonance is shown at each temperature increment. Samples clad in E7 and Lixon presented a thermal drift of 56.3 pm/°C and 52.3 pm/°C, respectively.

It can be seen in Figure 2 that the resonant wavelength shift of LC clad ring resonators is linear. This is to be expected, as the average LC index decreases linearly as temperature rises in both the anisotropic and isotropic phase [28,29].

**Figure 4.**
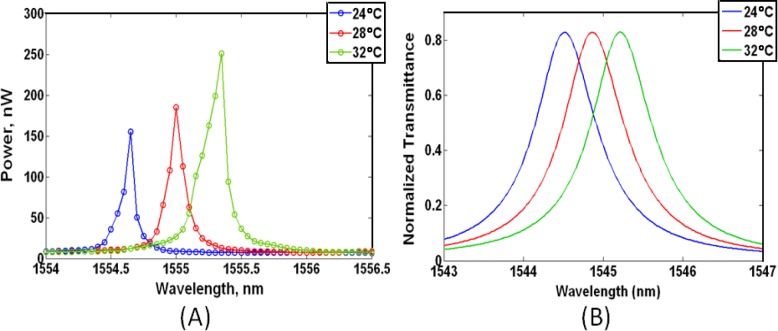
(**A**) Measured resonance shift in air of a ring resonator; (**B**) Simulated shift of a ring resonator clad in air as a function of rising temperature.

The measured resonance shifts were used in calculating the thermo-optic coefficients of the liquid crystal mixtures at 1550 nm. First, the measured air-clad ring resonator response served to validate a COMSOL Multiphysics model. The measured resonance shift of an air clad resonator was 87.5 pm/°C, which strongly agrees with the COMSOL simulation resonance shift of 87.5 pm/°C, and it gives us confidence in our model. The measured and the simulated results for an air cladding are depicted in Figure 4. Experimentally observed liquid crystal cladding resonance shifts were used to obtain the change in the silicon waveguide mode effective indices using Equation (1). The effective mode indices then served to calculate the thermo-optic coefficient of the LC mixtures using our COMSOL model. Table 3 contains a summary of the results. 5CB provides the greatest negative average refractive index <*n*> TOC of Δ*n*_5CB_/Δ*T* = −8.7 × 10^−4^/°C at 1550 nm. Lixon possesses the next best TOC of Δ*n*_Lixon_/Δ*T* = −7.2 × 10^−4^/°C; followed by E7 Δ*n*_E7_/Δ*T* = −6.7 × 10^−4^/°C, and MDA Δ*n*_MDA_/Δ*T* = −6.5 × 10^−4^/°C. In relation to literature, our estimated TOC value of E7 (Δ*n*_E7_/Δ*T* = −6.7 × 10^−4^/°C) is slightly greater than the average temperature dependent refractive index <*n*> value reported (Δ*n*/Δ*T* = −5.24 × 10^−4^/°C) [30]. This inconsistency is due to some of the E7 molecules being initially aligned in the *n*_e_ state as compared to a completely random <*n*> state. LC molecules in the *n*_e_ orientation will undergo a considerably larger refractive index change as a function of temperature in comparison to those randomly aligned [29,30]. It is not until the isotropic state that the LC Δ*n*/Δ*T* fully equalizes [29].

**Figure 5.**
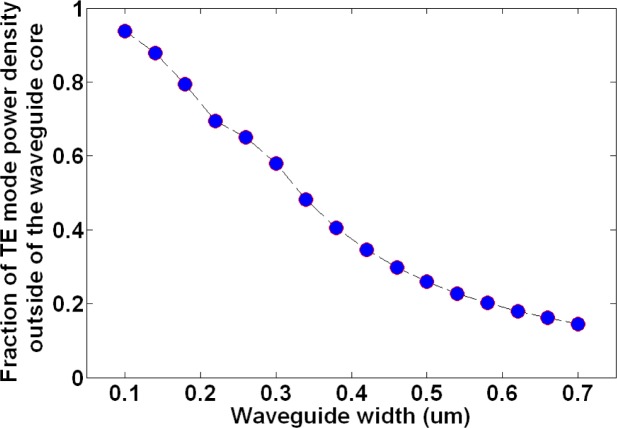
Silicon rectangular waveguide core width and the corresponding TE-like mode power density that extends into the cladding region. The waveguide height was kept constant at 250 nm. The cladding region refractive index was *n* = 1.53.

While liquid crystals aid in minimizing temperature associated effects, complete athermal response cannot be achieved without modifying the device geometry. The amount of thermal stabilization is directly related to the extent of the optical mode overlap with liquid crystals, or any other negative TOC material. For instance, the mode of narrower waveguides will sense more of the liquid crystal cladding due to a larger portion of it being present outside of the silicon core region. An example of this appears in Figure 5, which depicts the amount of TE like mode power density that extends outside of the waveguide core as a function of waveguide width. For a 500 nm wide, 250 nm tall silicon waveguide surrounded by *n* = 1.53 index cladding, a 26% mode overlap with the cladding region is achieved. A 300 nm wide waveguide will result in a 58% cladding overlap. It should be noted that, besides device geometry, the exact cladding material’s refractive index and surface roughness also come into play in mode confinement and propagation loss. Thus, while a narrower waveguide allows for increased interaction with the liquid crystal cladding, it also results in enhanced losses arising from a larger portion of the optical mode interacting with the sidewall surface roughness of the silicon core [31]. These interface imperfections originate during the fabrication process from line edge corrugations of the electron beam resist, pattern transfer, or from the etching process itself [32]. The roughness of core–cladding interfaces results in transmission loss that scales with the square of the roughness amplitude [32], which is the main reason why we chose waveguides of 500 nm width for our experiment as compared to narrower ones.

In our measurements, 5CB, which possesses the lowest clearing point temperature (*T*_ni_ = 35°C), boasted the largest negative TOC. 5CB is applicable to achieving predictable operation of silicon-based wavelength-division multiplexing (WDM) devices located on typical high performance multicore chips which may endure ±10 °C temperature variations [9]. Due to 5CB’s flash point of 113 °C [33], Lixon with its clearing point temperature of *T*_ni_ = 123 °C, may be better suited for applications in which the microprocessor die hot spot thermal range fluctuates between 70–120 °C [34].

## 3. Experimental

### 3.1. Sample Fabrication and Preparation

**Figure 6.**
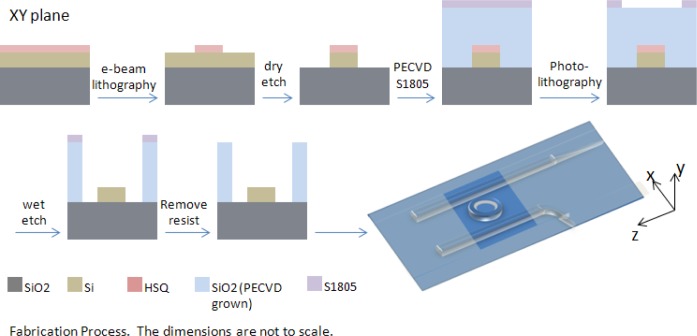
Fabrication process steps.

**Figure 7.**
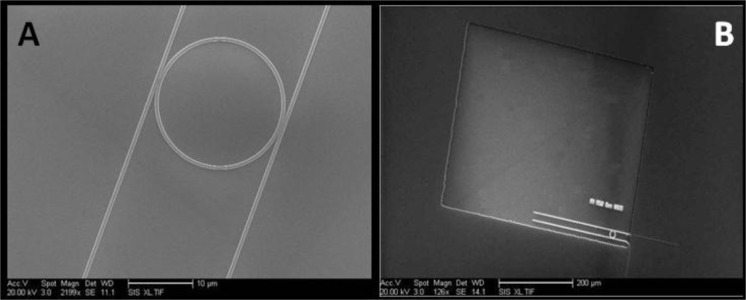
Fabricated ring resonator samples. (**A**) Close up of the ring resonator structure; (**B**) Ring resonator with a window etched in SiO_2_ for liquid crystals.

Samples were fabricated using a 680 μm thick silicon on insulator (SOI) wafer composed of a silicon handle, a 3 μm BOX layer and 250nm of silicon placed on top of the BOX. The 3 μm SiO_2_ layer aids in preventing the evanescent field of the optical mode from penetrating the silicon substrate below. Dow Corning (Midland, MI, USA) FOX-16 electron beam (e-beam) resist was diluted in Methyl isobutyl ketone (MIBK), one part FOX-16 to two parts MIBK (by weight), and spun at 4000 rpm resulting in a 180 nm thick coat [20]. The samples were patterned with a Vistec (Toronto, Canada) EBPG 5200 e-beam system using a dosage of 5120 μC/cm^2^ and developed in Tetramethylammonium hydroxide (TMAH) for 1 min. Dry etch of silicon was performed using Oxford Plasmalab 100 RIE/ICP (Oxford Instruments, Abingdon, Oxfordshire, UK) with a mixture of 25 sccm of SF_6_ and 50 sccm of C_4_F_8_ at a temperature of 15 °C, and with a reactive-ion etching (RIE) power of 30 W and inductively coupled plasma (ICP) power of 1200 W. The resulting silicon waveguides were covered by a 1800 nm layer of SiO_2_ cladding deposited via Oxford Plasmalab 80 Plus plasma-enhanced chemical vapor deposition (PECVD) at 350 °C using a mixture of 5% SiH_4_ and 95% N_2_ at 117 sccm with 710 sccm of N_2_O at a deposition rate of 72 nm/min. The PECVD chamber pressure was 1000 mT and the RF power was 20 W at 13.56 MHz. Window areas positioned over resonator rings were patterned with Shipley S1805 photoresist (Shipley Company, Marlborough, MA, USA), exposed in a Hybrid Technology Group (HTG) Mask Aligner and etched in a CMOS grade buffered oxide solution (BOE) consisting of 33.5% NH_4_, 7% HF, and 59.5% H_2_O, for a duration of 195 s. The remaining S1805 photoresist was removed with Shipley Microposit Remover 1165 (Shipley Company, Marlborough, MA, USA). The fabrication process is portrayed in Figure 6 and scanning electron microscope (SEM) images of the fabricated samples appear in Figure 7. Figure 7A depicts a silicon ring resonator of 9.9 μm radius and 500 nm width. Figure 7B shows the ring resonator sample clad in SiO_2_ and with a window etched over the ring to accommodate the LC cladding. Placement of the liquid crystal cladding was carried out in a clean room environment and preceded by a sample cleaning step using oxygen plasma. The oxygen plasma step aids in the removal of organic contaminants and it promotes adhesion and bonding to other surfaces.

### 3.2. Experimental Setup

**Figure 8.**
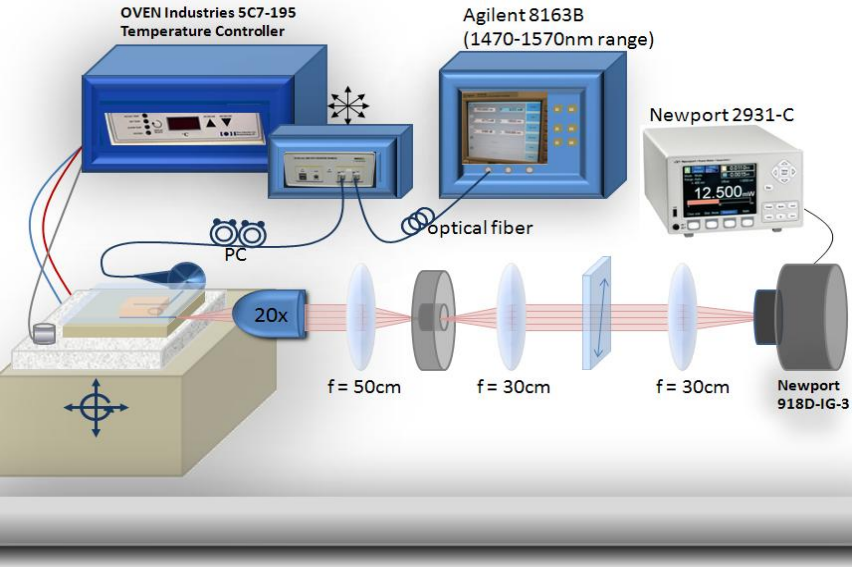
Optical setup.

Our experimental setup consisted of an Agilent 8163B telecom-grade tunable laser (Santa Clara, CA, USA) (1470–1570 nm range) connected to a polarization scrambler and fiber coupled to the on-chip waveguide. An Oven Industries 5C7-195 Benchtop Temperature Controller (Mechanicsburg, PA, USA) linked to a thermo-electric module assisted in the heating and cooling of the LC mixture. The thermoelectric module was embedded inside of a sample mount and the sample mount was placed on a three axis mechanical stage allowing for precise alignment with the input beam and imaging optics. A thermocouple fastened to the sample stage monitored the temperature to within 0.1 °C precision. The transmission spectrum was imaged in free space onto a Newport 2931-C power meter (Newport Corporation, Irvine, CA, USA). Control of the telecom source and the power meter was automated. The optical setup is illustrated in Figure 8.

## 4. Conclusions

In summary, we have explored the use of liquid crystals for passive temperature stabilization of silicon photonic devices. Liquid crystals possess high negative thermo-optic coefficients and their refractive index decreases linearly as temperature rises in both the anisotropic and isotropic phase. Using ring resonators clad in liquid crystals, we show that thermal drift can be mediated, although a full athermal response requires alteration of the silicon device geometry. The amount of thermal stabilization is directly related to the extent of the optical mode overlap with liquid crystals, or any other negative TOC material. The advantage of liquid crystals lies in their high negative thermo-optic coefficients in addition to low absorption at the infrared wavelengths.

## Figures and Tables

**Table 1.** Room temperature properties of liquid crystals used in the experiment.

Liquid Crystal Mixture	Clearing Point	Optical Anisotropy			
		Δ*n*	*n*_e_	*n*_o_	<*n*>
5CB (@22 °C, 589 nm)	35 °C	0.191	1.725	1.534	1.598
E7 (@20 °C, 589 nm)	58 °C	0.226	1.747	1.521	1.597
Lixon (@25 °C, 589 nm)	123 °C	0.109	1.596	1.487	1.523
MDA-05-2968 (@20 °C, 589 nm)	109.5 °C	0.2685	1.781	1.5125	1.602

**Table 2.** Summary of results appearing in Figure 2.

Liquid Crystal Cladding	Resonance shift/°C	Measured Temperature Range
5CB	40 pm	24 – 32 °C
E7	56.3 pm	24 – 56 °C
MDA-05-2968	58 pm	24 – 74 °C
Lixon	52.3 pm	24 – 46 °C

**Table 3.** Change in the effective index as a function of a 30 °C increase in temperature and the corresponding thermo-optic coefficient of the liquid crystal mixture at 1550 nm.

Liquid crystal cladding	Effective index shift for a 30 °C rise in temperature	LC Δ*n*/Δ*T*
5CB	Δ*n*_eff_ = −0.0021	−0.00087/°C
E7	Δ*n*_eff_ = −0.0029	−0.00067/°C
Lixon	Δ*n*_eff_ = −0.0027	−0.00072/°C
MDA-05-2968	Δ*n*_eff_ = −0.0030	−0.00065/°C
